# Computed Tomographic Evaluation of the Morphometry of the Intervertebral Foramina and Intervertebral Discs of the Cervical Spine

**DOI:** 10.7759/cureus.96461

**Published:** 2025-11-09

**Authors:** Phanindra P Poudel, Chacchu Bhattarai, Nishant Pandey, Prakash Sharma

**Affiliations:** 1 Anatomy, Western Regional Hospital, Pokhara Academy of Health Sciences, Pokhara, NPL; 2 Anatomy, Manipal College of Medical Sciences, Pokhara, NPL; 3 Radiology, Manipal College of Medical Sciences, Pokhara, NPL

**Keywords:** cervical spine, computed tomography, intervertebral disc, intervertebral foramen, morphometry

## Abstract

Background: The cervical spine comprises vertebrae and intervertebral discs that facilitate neck mobility and protect neurovascular structures. Intervertebral foramina serve as conduits for spinal nerves and vessels and may undergo morphological changes with aging and degeneration, potentially leading to neurological symptoms. The objectives of this study included performing a CT analysis of the morphometry of intervertebral foramina and the height of the intervertebral disc across different levels of the cervical spine, with comparisons based on age, vertebral level, gender, and laterality.

Methods: This observational study analyzed CT scans of the cervical spine from 104 patients. Vertical height, anteroposterior depth, and cross-sectional area of intervertebral foramina were measured bilaterally at levels C2C3 to C6C7. Intervertebral disc height was also assessed. Participants were categorized into four age groups and by sex. Statistical analyses included one-way ANOVA, paired samples t-test, Pearson correlation coefficient, and one-sample t-test.

Results: Foraminal dimensions, especially vertical height and cross-sectional area, showed a statistically significant decline with increasing age, most prominently at lower cervical levels (C5C6 and C6C7). Intervertebral disc height also decreased significantly with age, particularly at C5C6. Males demonstrated consistently larger foraminal dimensions and disc heights compared to females. Except for C4C5, foraminal dimensions were largely symmetrical between right and left sides. Age-related degenerative changes in the cervical spine predominantly affect the lower cervical levels, leading to a reduction in foraminal size and disc height.

Conclusion: The observed morphometric variations, alongside gender differences, underscore the importance of individualized anatomical assessment in the diagnosis and management of cervical spine pathologies.

## Introduction

The cervical spine comprises seven cervical vertebrae and the intervertebral discs between them [1]. The intervertebral foramen is a passage between adjacent cervical vertebrae connecting the spinal canal and the extraspinal region [1,2]. The intervertebral foramen, also called the neural foramen, is bounded by adjacent vertebral pedicles superiorly and inferiorly, anteriorly by the uncinate process, intervertebral disc, and inferior portion of the vertebral body, and posteriorly by the facet joint and superior articular process [1,2]. It carries anterior and posterior nerve roots with the posterior root ganglion, along with lymphatics, blood vessels, and areolar and fatty tissue [3,4]. Intervertebral discs have a soft nucleus pulposus surrounded by a cartilaginous ring of annulus fibrosus [5,6]. They support and provide cushioning and elasticity to the spine [5].

Morphometric knowledge of the intervertebral foramen can aid in diagnosing foraminal stenosis of the cervical spine in patients with cervical spondylosis and radiculopathy [7]. Cervical radiculopathy, often presenting with neck and arm pain, is typically caused by compression of the cervical nerve root by a herniated disc or bony osteophytes, most commonly affecting the C7 root (C6C7 herniation), followed by C6 (C5C6 herniation) and C8 (C7T1 herniation) nerve roots [8]. Radiologists need to understand the vascularization of the spinal cord and the origin of the arteries supplying it, which occurs along the intervertebral foramen, to avoid iatrogenic complications during interventional procedures [1]. Foraminal stenosis is also caused by osteophytosis of the uncovertebral joint projecting into the foramen [9,10]. Intervertebral foramen stenosis has been implicated in conditions such as postoperative C5 palsy and transient neurapraxia among athletes [11].

The cervical spine often undergoes degenerative changes as a natural part of aging [7]. Such changes can lead to disc misalignment, displacement, calcification, and ossification along with spinal stenosis, causing impairment of cervical nerve roots [7,12]. Cervical intervertebral disc calcification in children can cause transient symptoms such as pain, stiffness, limited motion, fever, and torticollis [12]. Major degenerative disorders of intervertebral discs include intervertebral (osteo)chondrosis and spondylosis deformans [12,13]. Intervertebral disc protrusion deforms the dural tube, causing spinal canal stenosis [9].

Advanced imaging techniques such as computed tomography myelography (CTM) can accurately measure the size of the spinal cord and canal, helping determine the severity of conditions like compression myelopathy [14]. Although magnetic resonance imaging (MRI) is considered the gold standard for diagnosing soft tissue pathologies, computed tomography (CT) remains a commonly used technique to detect bony abnormalities [7]. This study describes the morphometric analysis of the intervertebral foraminal area and the thickness of intervertebral discs in the cervical spine through evaluation of CT scan images.

## Materials and methods

An observational (descriptive) study was conducted on patients undergoing CT scans of the head and neck region at Manipal Teaching Hospital, Manipal College of Medical Sciences, Pokhara, Nepal, over a period of more than three years, from February 2022 to April 2025. A non-probability convenience sampling method was used. The total sample size was 104, determined pragmatically based on available resources and the number of eligible subjects accessible during the study period. The inclusion criteria were CT scan radiographic images (128-slice machine, Philips) that clearly covered the entire cervical spine (C1-C7) with good image quality, including both adults and adolescents, and with bilateral visualization of the intervertebral foramina and discs. Only scans performed using a standard high-resolution CT protocol were included. The exclusion criteria were scans from patients with a history of cervical spine surgery, instrumentation, or radiation; those with acute trauma or congenital anomalies associated with cervical spine development, such as hemivertebrae and spina bifida; or major cervical pathology, including tumors, abscesses, or gross deformities. Cases with severe inflammatory or metabolic bone disease affecting vertebral anatomy were also excluded, as were scans that were incomplete, of poor quality, or affected by artifacts. Potential confounding variables included differences in age and sex, variations in body size (height, weight, BMI), and the presence of degenerative changes such as disc degeneration, osteophytes, or spondylosis. Additional factors that could influence the results included scanner model, slice thickness, technical parameters, observer measurement variability, and anatomical variations related to ethnicity or population.

**Figure 1:**
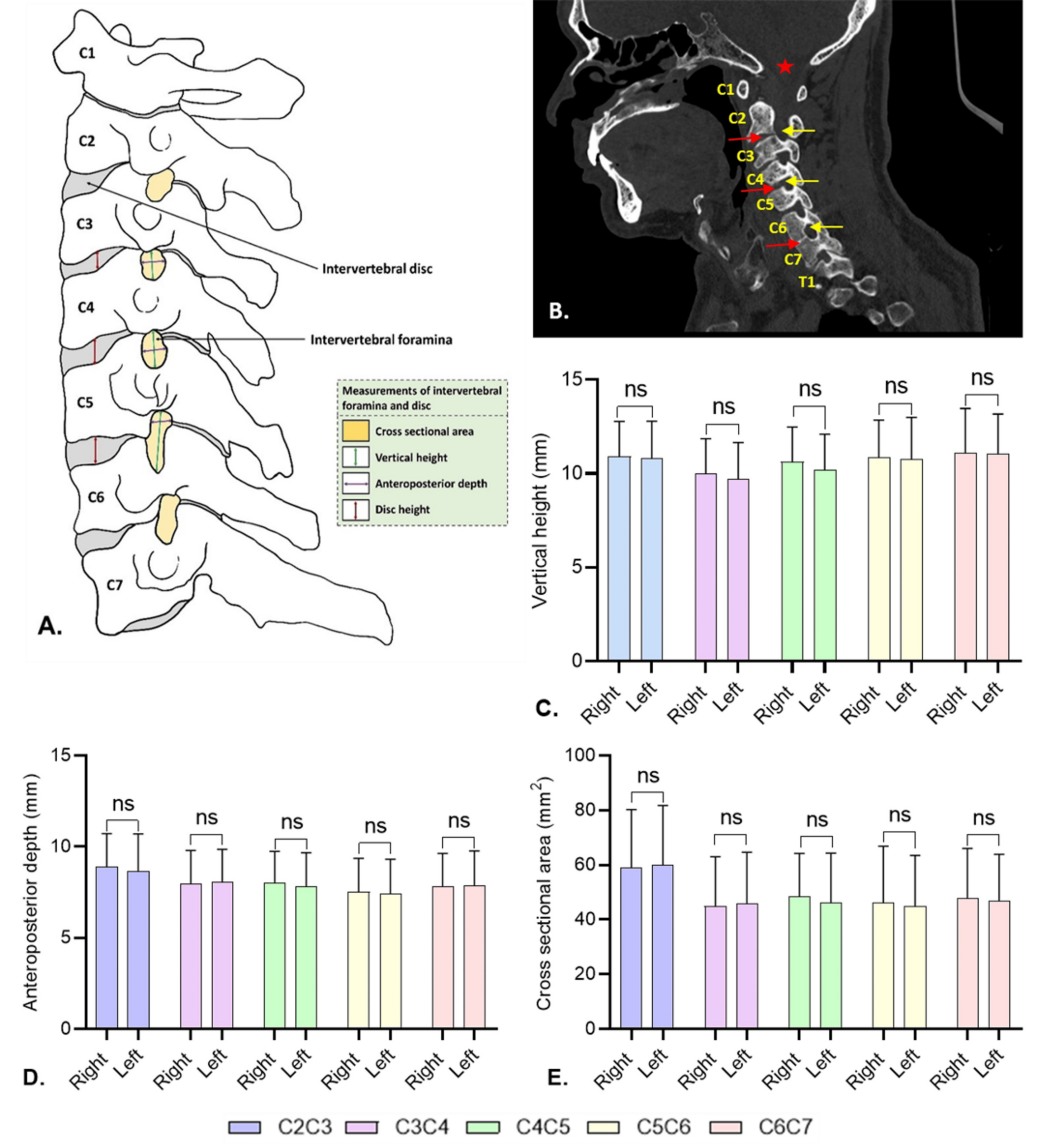
Cervical spine and comparison of right- and left-sided intervertebral foraminal vertical height, anteroposterior depth, and cross-sectional area using paired-samples t-test (A) Schematic illustration showing the lateral view of the cervical spine (original work of the author). (B) Sagittal CT image of the cervical spine of a 59-year-old male taken at 1.45× zoom. (C–E) Comparison of right- and left-sided intervertebral foraminal measurements at various vertebral levels using paired-samples t-test (ns = not significant). (C) Vertical height. (D) Anteroposterior depth. (E) Cross-sectional area. C, cervical vertebrae; T, thoracic vertebrae. In (B), the red star represents the foramen magnum; yellow arrows represent intervertebral foramina at various levels; red arrows represent intervertebral discs at multiple levels.

Measurements, including vertical height (VH), anteroposterior depth (APD), and cross-sectional area (CA) of the intervertebral foramina (Figure 1A, B) at different cervical levels, were carried out bilaterally. All scale data (VH, APD, and CA) of intervertebral foramina and disc height (DH) of the intervertebral disc were presented as mean ± standard deviation (SD). Data analysis was conducted to identify significant differences based on age, vertebral level, gender, and laterality, using statistical analyses including one-way ANOVA, paired-samples t-test, Pearson correlation coefficient, and one-sample t-test (P ≤ 0.05) in IBM SPSS Statistics for Windows, Version 26 (Released 2019; IBM Corp., Armonk, New York) and Jamovi 2.3.13 software (The jamovi Project, Sydney, Australia). GraphPad Prism 8.4.2 software (GraphPad Software, Inc., San Diego, California) was used for graph plotting. The study population was categorized into four distinct age groups to reflect different stages of life: adolescents (<19 years), adults (19 to <25 years), seniors (25-64 years), and elders (>64 years). Adolescents represent the transition from childhood to adulthood, characterized by rapid physical, emotional, and social development. Adults correspond to the phase of emerging adulthood, a period of forming independent identities, making important life decisions, and beginning career exploration. Seniors typically represent the working-age population, often engaged in career building, family responsibilities, and financial planning. Elders represent the later stage of life, a period that may include retirement, increased leisure, and age-related health challenges. In addition to age, data were also classified by gender, including both male and female participants (Table 1).

**Table 1:** Demographic characteristics of the cohort

Characteristic	Details
Sample size	104 patients (CT scans)
Age groups	Adolescents (<19 yrs)
	Adults (19–<25 yrs)
	Seniors (25–64 yrs)
	Elders (>64 yrs)
Sex	Male: 56 (53.8%)
	Female: 48 (46.2%)
Study site	Manipal Teaching Hospital, Pokhara, Nepal
Imaging modality	128 Slice Machine, Phillips

## Results

Age-wise comparison of intervertebral foraminal size at different levels of the cervical spine

Table 2 shows a decline in foraminal size with increasing age across multiple parameters, particularly in the right and left vertical heights and the right and left cross-sectional areas. Several parameters demonstrate statistically significant age-related changes (P<0.05), indicating meaningful differences across age groups. The most significant reductions occur at the lower cervical levels. However, the upper cervical levels do not show significant differences (P>0.05), suggesting greater stability with aging. 

**Table 2:** Age-wise comparison of intervertebral foraminal size at different levels of cervical spine C: cervical vertebrae, RVH: right vertical height, LVH: left vertical height, RAPD: right anteroposterior depth, LAPD: left anteroposterior depth, RCA: right cross-sectional area, LCA: left cross-sectional area. ^a^P<0.05, ^b^P<0.01, ^c^P<0.0001.

Intervertebral foraminal parameters at different vertebral levels (n=104)	Adolescents (<19 years) (mean±SD), mm or mm^2^	Adults (19 to <25 years) (mean±SD), mm or mm^2^	Seniors (25-64 years) (mean±SD), mm or mm^2^	Elders (>64 years) (mean±SD), mm or mm^2^	One-way ANOVA test
C2C3RVH	12.13±2.36	11.43±1.27	10.89±2.01	10.65±1.7	0.8 (P=0.49)
C3C4RVH	11.2±1.13	11.02±1.7	10.09±1.89	9.3±1.77	3.11 (P=0.05^a^)
C4C5RVH	12.6±3.18	11.73±1.85	10.51±1.7	10.34±1.95	1.38 (P=0.06)
C5C6RVH	11.6±4.84	12.16±2.07	11.01±1.84	10.14±1.76	2.36 (P=0.04^a^)
C6C7RVH	11.73±4.02	12.61±2.2	11.45±2.38	9.86±1.66	5.52 (P=0.004^b^)
C2C3LVH	11.1±4.12	11.68±1.83	10.76±2.1	10.56±1.63	0.71 (P=0.57)
C3C4LVH	10.96±4.37	10.93±1.5	9.84±1.8	9.00±1.82	2.88 (P=0.03^a^)
C4C5LVH	11.2±3.72	11.37±2.3	10.45±1.77	9.09±1.51	4.99 (P=0.002^b^)
C5C6LVH	12±2.29	12.42±2.08	11.04±2.08	9.55±2.08	4.76 (P=0.001^b^)
C6C7LVH	12.7±3.01	11.87±2.5	11.24±1.96	10.27±2.08	1.8 (P=0.05^a^)
C2C3RAPD	8.73±3.61	8.68±1.61	8.9±1.75	9.03±1.9	0.08 (P=0.96)
C3C4RAPD	7.83±1.8	7.96±2.24	7.98±1.9	7.96±11.65	0.005 (P=0.99)
C4C5RAPD	7.76±2.48	7.26±1.67	8.10±1.7	8.08±1.78	0.53 (P=0.62)
C5C6RAPD	8.43±2.15	7.57±1.97	7.88±1.82	6.58±1.47	4.02 (P=0.01^a^)
C6C7RAPD	8.06±1.44	8.26±1.56	8.11±1.77	6.89±1.9	2.61 (P=0.02^a^)
C2C3LAPD	8.86±2.78	8.41±2.04	8.66±1.98	8.72±2.26	0.04 (P=0.98)
C3C4LAPD	7.9±1.61	7.61±2.19	8.26±1.85	7.72±1.68	0.64 (P=0.52)
C4C5LAPD	9.16±3.52	7.68±2.37	8.04±1.77	7.14±1.66	1.7 (P=0.1)
C5C6LAPD	8.4±3.24	8.08±1.9	7.55±1.75	6.78±2.03	1.24 (P=0.15)
C6C7LAPD	9.26±0.7	8.51±1.87	8.04±1.87	7.11±1.95	4.69 (P=0.05^a^)
C2C3RCA	83.13±35.17	57.28±21.01	59.91±21.37	55.16±18.45	0.76 (P=0.17)
C3C4RCA	51.43±25.08	47.83±14.46	46.06±19.07	40.58±16.09	0.78 (P=0.48)
C4C5RCA	57.90±17.76	45.35±19.65	50.12±16.01	44.44±13.27	1.2 (P=0.26)
C5C6RCA	57.7±28.08	49.52±19.6	50.87±20.6	33.6±14.49	6.54 (P=0.001^b^)
C6C7RCA	50.6±21.11	58.35±14.42	51.8±17.06	35.77±15.82	6.89 (P=0.0^c^)
C2C3LCA	75.63±46.79	54.36±25.27	61.97±22.46	55.23±14.63	1.01 (P=0.26)
C3C4LCA	70.93±32.59	49.78±20.97	48.22±17.85	37.01±14.67	3.59 (P=0.003^b^)
C4C5LCA	58.56±31.01	46.46±19.32	48.93±17.29	38.64±16.59	2.23 (P=0.04^a^)
C5C6LCA	54.36±27.69	45.18±16.96	48.81±17.93	34.61±16.43	4.07 (P=0.005^b^)
C6C7LCA	65.6±23.46	49.73±18.8	49.11±15.04	39.2±17.51	2.53 (P=0.01^a^)

Level-wise comparison of intervertebral foraminal vertical height

Table 3 presents a pairwise comparison of intervertebral foraminal vertical height (VH) across different cervical spine levels on both the right and left sides using paired-samples t-test and Pearson correlation coefficient. All pairs demonstrate moderate to strong positive correlations (0.41 to 0.7), all highly significant (P=0.0), indicating that adjacent levels tend to follow a similar trend as the foraminal height increases or decreases at one level. Significant level-wise differences in foraminal vertical height are observed, particularly between C3C4 and other levels on both sides. C5C6 and C6C7 show greater consistency, with fewer significant changes, suggesting relative anatomical stability in foraminal height at these levels. These findings may have clinical relevance in understanding age-related or pathological changes in cervical foramina and their potential impact on nerve root compression.

**Table 3:** Level-wise comparison of intervertebral foraminal vertical height C: cervical vertebrae. ^a^P<0.05, ^b^P<0.01, ^c^P<0.0001.

Variables	Category	(Mean±SD), mm	Paired samples t-test	Pearson correlation coefficient
Right side (mm) (n=104)				
C2C3 (10.9±1.88)	C3C4	9.98±1.87	5.36 (P=0.0^d^)	0.57 (P=0.0^d^)
	C4C5	10.62±1.86	1.54 (P=0.12)	0.51 (P=0.0^d^)
	C5C6	10.88±1.98	0.11 (P=0.9)	0.49 (P=0.0^d^)
	C6C7	11.12±2.35	-1.02 (P=0.3)	0.47 (P=0.0^d^)
C3C4 (9.98±1.87)	C4C5	10.62±1.86	-3.9 (P=0.0^d^)	0.6 (P=0.0^d^)
	C5C6	10.88±1.98	-4.72 (P=0.0^d^)	0.5 (P=0.0^d^)
	C6C7	11.12±2.35	-5.48 (P=0.0^d^)	0.48 (P=0.0^d^)
C4C5 (10.62±1.86)	C5C6	10.88±1.98	-1.4 (P=0.16)	0.53 (P=0.0^d^)
	C6C7	11.12±2.35	-2.33 (P=0.02^a^)	0.48 (P=0.0^c^)
C5C6 (10.62±1.86)	C6C7	11.12±2.35	-1.37 (P=0.17)	0.66 (P=0.0^c^)
Left side (mm) (n=104)				
C2C3 (10.79±2.01)	C3C4	9.73±1.92	5.86 (P=0.0^c^)	0.56 (P=0.0^c^)
	C4C5	10.18±1.92	3.15 (P=0.002^b^)	0.49 (P=0.0^c^)
	C5C6	10.77±2.22	0.07 (P=0.94)	0.41 (P=0.0^c^)
	C6C7	11.07±2.1	-1.29 (P=0.19)	0.41 (P=0.0^c^)
C3C4 (9.73±1.92)	C4C5	10.18±1.92	-2.91 (P=0.004^b^)	0.67 (P=0.0^c^)
	C5C6	10.77±2.22	-5.75 (P=0.0^c^)	0.61 (P=0.0^c^)
	C6C7	11.07±2.1	-6.54 (P=0.0^c^)	0.46 (P=0.0^c^)
C4C5 (10.18±1.92)	C5C6	10.77±2.22	-3.72 (P=0.0^c^)	0.7 (P=0.0^c^)
	C6C7	11.07±2.1	-4.75 (P=0.0^c^)	0.55 (P=0.0^c^)
C5C6 (10.77±2.22)	C6C7	11.07±2.1	-1.51 (P=0.56)	0.13 (P=0.0^c^)

Level-wise comparison of intervertebral foraminal anteroposterior depth

Table 4 compares the anteroposterior depth (APD) of the intervertebral foramina at different cervical levels on the right and left sides in 104 subjects using paired-samples t-test and Pearson correlation coefficient. Significant variations in APD are observed, particularly from C2C3 downward (notably on the left side) and between C4C5 and C5C6, consistently on both sides. C5C6 and C6C7 tend to show more stable or smaller variations. These findings may have implications for understanding cervical spine degeneration patterns, assessing nerve compression risk, and planning surgical decompression procedures.

**Table 4:** Level-wise comparison of intervertebral foraminal anteroposterior depth C: cervical vertebrae. ^a^P<0.05, ^b^P<0.01, ^c^P<0.0001.

Variables	Category	(Mean±SD), mm	Paired samples t-test	Pearson correlation coefficient
Right side (mm) (n=104)				
C2C3 (8.91±1.81)	C3C4	7.96±1.83	6.18 (P=0.0^c^)	0.63 (0.0^c^)
	C4C5	8.02±1.72	5.7 (P=0.12)	0.59 (0.0^c^)
	C5C6	7.52±1.82	7.83 (P=0.9)	0.5 (0.0^c^)
	C6C7	7.79±1.84	5.35 (P=0.3)	0.31 (0.001^b^)
C3C4 (7.96±1.83)	C4C5	8.02±1.72	-0.38 (P=0.69)	0.66 (0.0^c^)
	C5C6	7.52±1.82	2.61 (P=0.01^a^)	0.55 (0.0^c^)
	C6C7	7.79±1.84	0.94 (P=0.34)	0.46 (0.0^c^)
C4C5 (8.02±1.72)	C5C6	7.52±1.82	3.03 (P=0.003^b^)	0.56 (0.0^c^)
	C6C7	7.79±1.84	1.19 (P=0.23)	0.39 (0.0^c^)
C5C6 (7.52±1.82)	C6C7	7.79±1.84	-1.59 (P=0.11)	0.57 (0.0^c^)
Left side (mm) (n=104)				
C2C3 (8.66±2.05)	C3C4	8.05±1.81	3.46 (P=0.001^b^)	0.57 (0.0^c^)
	C4C5	7.80±1.87	4.33 (P=0.0^c^)	0.46 (0.0^c^)
	C5C6	7.41±1.9	6.07 (P=0.0^c^)	0.43 (0.0^c^)
	C6C7	7.86±1.91	3.7 (P=0.0^c^)	0.37 (0.001^b^)
C3C4 (8.05±1.81)	C4C5	8.02±1.72	1.84 (P=0.06)	0.7 (0.0^c^)
	C5C6	7.41±1.9	3.57 (P=0.001^b^)	0.51 (0.0^c^)
	C6C7	7.86±1.91	1.14 (P=0.25)	0.56 (0.0^c^)
C4C5 (7.80±1.87)	C5C6	7.41±1.9	2.47 (P=0.01^a^)	0.63 (0.0^c^)
	C6C7	7.86±1.91	-0.33 (P=0.74)	0.54 (0.0^c^)
C5C6 (7.41±1.90)	C6C7	7.86±1.91	-2.57 (P=0.01^a^)	0.56 (0.0^c^)

Level-wise comparison of intervertebral foraminal cross-sectional area

This study examines the variation in intervertebral foraminal cross-sectional area (CA) at cervical spine levels C2C3 through C6C7 on both the right and left sides in 104 subjects using paired-samples t-test and Pearson correlation coefficient (Table 5). The intervertebral foraminal CA decreases significantly from C2C3 to the lower cervical levels on both sides, with the most pronounced reduction between C2C3 and C3C4. However, the foraminal areas from C3C4 to C6C7 show no statistically significant differences, indicating anatomical consistency in the lower cervical segments. High interlevel correlations suggest predictable morphological trends, which are valuable for radiologic assessment and surgical planning.

**Table 5:** Level-wise comparison of intervertebral foraminal cross-sectional area C: cervical vertebrae. ^a^P<0.01, ^b^P<0.0001.

Variables	Category	(Mean±SD), mm^2^	Paired samples t-test	Pearson correlation coefficient
Right side (mm^2^) (n=104)				
C2C3 (59.10±21.18)	C3C4	44.88±18.11	7.28 (P=0.0^b^)	0.49 (0.0^b^)
	C4C5	48.45±15.72	5.78 (P=0.0^b^)	0.51 (0.0^b^)
	C5C6	46.31±20.53	6.14 (P=0.0^b^)	0.48 (0.0^b^)
	C6C7	47.96±18.1	4.95 (P=0.0^b^)	0.32 (0.001^a^)
C3C4 (44.88±18.11)	C4C5	48.45±15.72	-2.65 (P=0.009^a^)	0.67 (0.0^b^)
	C5C6	46.31±20.53	-0.82 (P=0.41)	0.58 (0.0^b^)
	C6C7	47.96±18.1	-1.78 (P=0.07)	0.53 (0.0^b^)
C4C5 (48.45±15.72)	C5C6	46.31±20.53	1.35 (P=0.17)	0.63 (0.0^b^)
	C6C7	47.96±18.1	0.28 (P=0.77)	0.46 (0.0^b^)
C5C6 (46.31±20.53)	C6C7	47.96±18.1	-1.19 (P=0.23)	0.74 (0.0^b^)
Left side (mm^2^) (n=104)				
C2C3 (59.96±21.76)	C3C4	45.98±18.68	9.21 (P=0.0^b^)	0.71 (0.0^b^)
	C4C5	46.25±18.07	8.84 (P=0.0^b^)	0.7 (0.0^b^)
	C5C6	44.87±18.6	8.28 (P=0.0^b^)	0.58 (0.0^b^)
	C6C7	46.96±16.9	6.39 (P=0.0^b^)	0.44 (0.0^b^)
C3C4 (45.98±18.68)	C4C5	46.25±18.07	-0.19 (P=0.84)	0.71 (0.0^b^)
	C5C6	44.87±18.6	0.73 (P=0.46)	0.66 (0.0^b^)
	C6C7	46.96±16.9	-0.63 (P=0.52)	0.61 (0.0^b^)
C4C5 (46.25±18.07)	C5C6	44.87±18.6	1.03 (P=0.3)	0.72 (0.0^b^)
	C6C7	46.96±16.9	-0.44 (P=0.65)	0.55 (0.0^b^)
C5C6 (44.87±18.60)	C6C7	46.96±16.9	-1.36 (P=0.17)	0.61 (0.0^b^)

Comparison of right and left-sided intervertebral foraminal measurements

Figure 1C to E compares the right and left intervertebral foraminal measurements, including VH, APD, and CA, across cervical vertebral levels C2C3 to C6C7 in 104 individuals using paired-samples t-test. The only significant asymmetry was observed in vertical height at C4C5, favoring the right side. Overall, the intervertebral foraminal dimensions are largely symmetrical between the right and left sides of the cervical spine.

Gender-wise comparison of intervertebral foraminal size at different vertebral levels of the cervical spine

**Figure 2:**
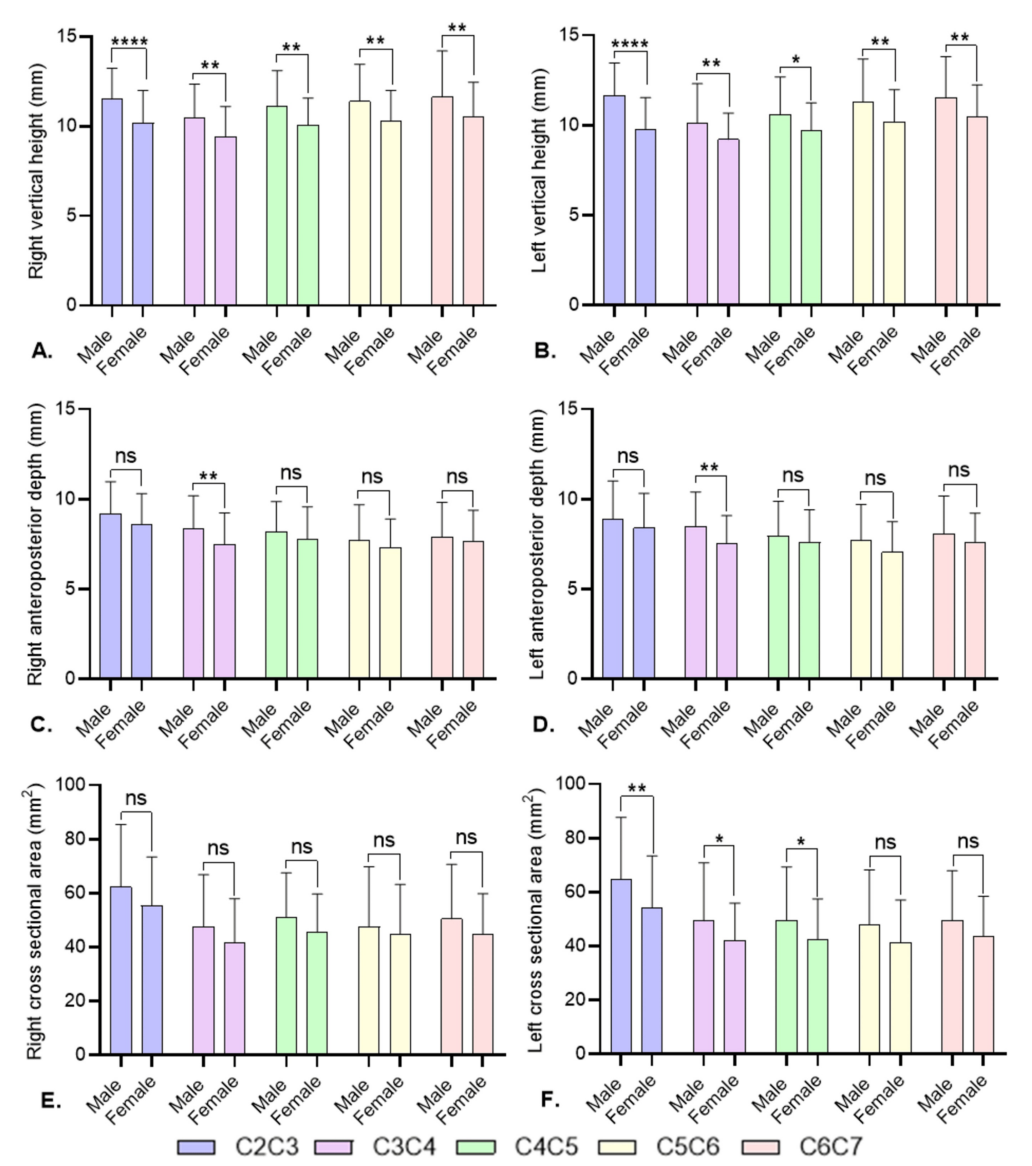
Gender-wise comparison of intervertebral foraminal size at different vertebral levels of the cervical spine using independent samples t-test (A) Right vertical height, (B) left vertical height, (C) right anteroposterior depth, (D) left anteroposterior depth, (E) right cross-sectional area, and (F) left cross-sectional area. The results are presented as mean±SD. *P<0.05, **P<0.01, ****P<0.0001. ns: not significant, C: cervical vertebrae.

Figure 2 compares intervertebral foraminal measurements between males and females at various cervical levels using independent-samples t-test. Three main parameters were evaluated: VH, APD, and CA. Males consistently demonstrated larger intervertebral foraminal vertical height at all levels. Some levels showed greater anteroposterior depth and cross-sectional area in males, particularly at C3C4 and C4C5, although the differences were not uniform across all parameters or levels. These findings suggest sexual dimorphism in cervical foraminal dimensions, with males generally having larger foramina.

Age-wise comparison of intervertebral disc height at different levels of the cervical spine

Disc height generally decreases with age across most cervical levels (Table 6). The mean overall IVDH (IVDHM) shows a significant decrease with age. C5C6 level exhibits the most notable and significant age-related reduction in disc height, with elders having the lowest value. Other levels (C2C3, C3C4, C4C5, C6C7) showed no significant differences, although the trend still suggests decreasing disc height with advancing age. Cervical intervertebral disc height declines progressively with age, with the C5C6 level most affected. This supports the association between aging and degenerative disc changes, particularly in the lower cervical spine.

**Table 6:** Age-wise comparison of intervertebral disc height at different levels of cervical spine ^a^P<0.05, ^b^P<0.01. C: cervical vertebrae, IVDH: intervertebral disc height, IVDHM: mean intervertebral disc height.

Intervertebral disc height at different vertebral levels (n=104)	Adolescents (<19 years) (mean±SD), mm	Adults (19-<25 years) (mean±SD), mm	Seniors (25-64 years) (mean±SD), mm	Elders (>64 years) (mean±SD), mm	One-way ANOVA test
C2C3IVDH	6.56±0.4	5.76±1.28	5.42±1.07	5.31±1.01	1.47 (P=0.22)
C3C4IVDH	6.63±0.56	5.8±1.26	5.84±1.02	5.28±1.34	2.32 (P=0.07)
C4C5IVDH	6.63±1.11	5.77±1.34	5.61±1.05	5.27±1.45	1.45 (P=0.23)
C5C6IVDH	6.5±1.6	5.92±1.27	5.55±1.31	4.53±1.49	4.93 (P=0.003^b^)
C6C7IVDH	6.96±1.15	5.95±1.18	6.06±1.22	5.38±1.54	2.38 (P=0.07)
IVDHM	6.66±0.92	5.84±1.16	5.7±0.95	5.16±1.04	3.31 (P=0.02^a^)

Gender-wise comparison of intervertebral disc height at different levels of the cervical spine

**Figure 3:**
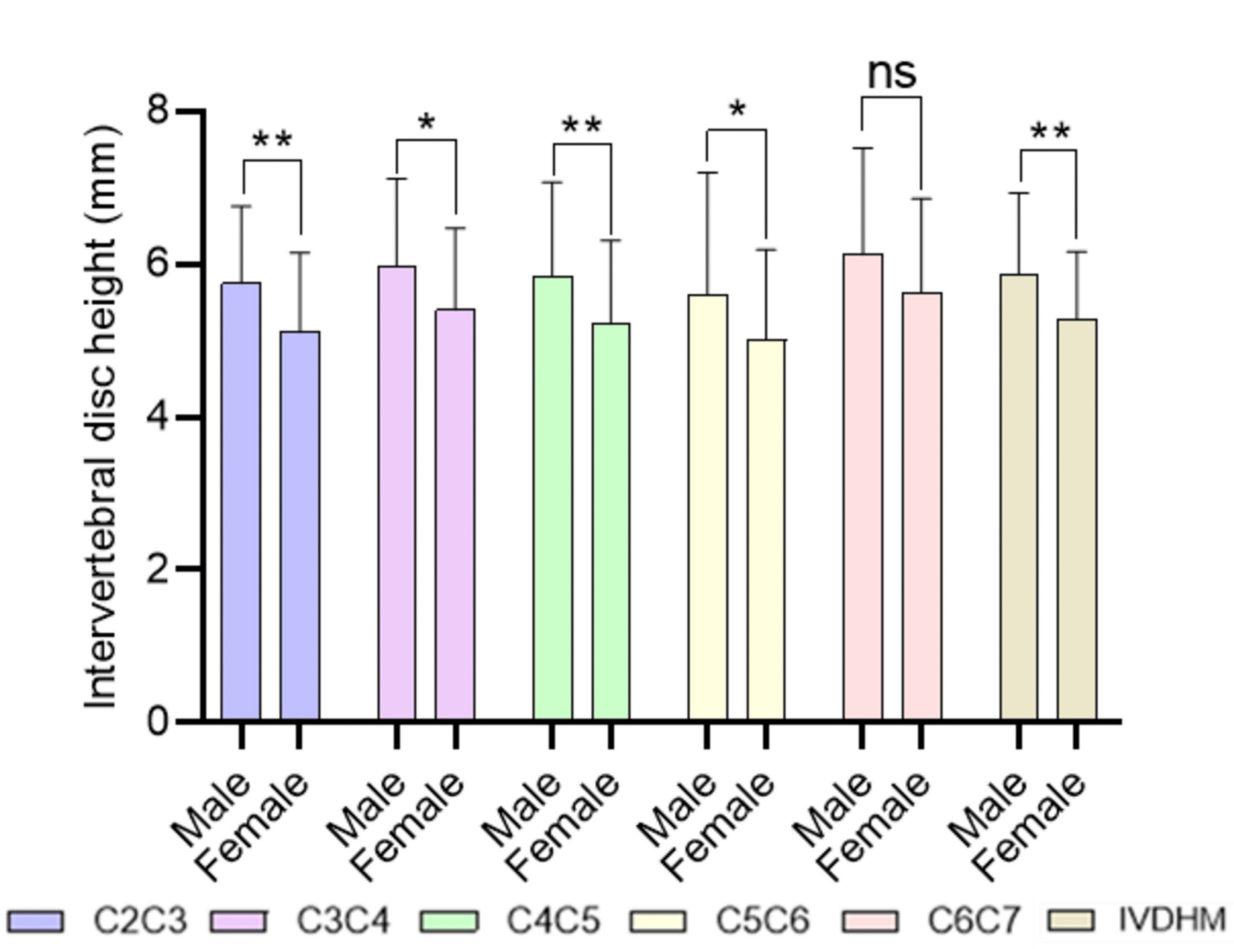
Gender-wise comparison of intervertebral disc height at different levels of the cervical spine using independent samples t-test The results are presented as mean±SD. *P<0.05, **P<0.01. ns: not significant, C: cervical vertebrae.

This comparison (Figure 3) evaluates intervertebral disc height between male (n=56) and female (n=48) participants across various cervical spine levels using independent-samples t-test. A gender-based difference in cervical disc height is observed, with males having significantly greater intervertebral disc heights at most levels. This finding may reflect underlying anatomical or biomechanical differences between the sexes. 

Average value of intervertebral foramina and intervertebral disc measurements at different levels of the cervical spine

The cervical intervertebral foramina show vertical heights averaging around 10-11 mm, which are greater than their anteroposterior depths of about 7-9 mm. The cross-sectional area is largest at the upper cervical level (C2C3, approximately 60 mm²) and decreases toward the mid-cervical region (C4-C6, approximately 45-48 mm²). Intervertebral disc height remains relatively uniform across levels, averaging 5-6 mm. Overall, the foramina are taller than they are deep, with disc height showing only minor variation (Table 7).

**Table 7:** Average value of intervertebral foramina and intervertebral disc measurements at different vertebral levels ^d^P<0.0001. C: cervical vertebrae, RVH: right vertical height, LVH: left vertical height, RAPD: right anteroposterior depth, LAPD: left anteroposterior depth, RCA: right cross-sectional area, LCA: left cross-sectional area.

Intervertebral foraminal and disc measurements at different vertebral levels (n=104)	(Mean±SD), mm or mm^2^	One-sample t-test
C2C3RVH	10.9±1.88	58.91 (P=0.0^a^)
C3C4RVH	9.98±1.87	54.3 (P=0.0^a^)
C4C5RVH	10.62±1.86	58.19 (P=0.0^a^)
C5C6RVH	10.88±1.98	55.8 (P=0.0^a^)
C6C7RVH	11.12±2.35	48.16 (P=0.0^a^)
C2C3LVH	10.79±2.01	54.56 (P=0.0^a^)
C3C4LVH	9.73±1.92	51.57 (P=0.0^a^)
C4C5LVH	10.18±1.92	54.02 (P=0.0^a^)
C5C6LVH	10.77±2.22	49.46 (P=0.0^a^)
C6C7LVH	11.07±2.1	53.63 (P=0.0^a^)
C2C3RAPD	8.91±1.81	50.15 (P=0.0^a^)
C3C4RAPD	7.96±1.83	44.24 (P=0.0^a^)
C4C5RAPD	8.02±1.72	47.35 (P=0.0^a^)
C5C6RAPD	7.52±1.82	42.15 (P=0.0^a^)
C6C7RAPD	7.79±1.84	43.14 (P=0.0^a^)
C2C3LAPD	8.66±2.05	43.08 (P=0.0^a^)
C3C4LAPD	8.05±1.81	45.17 (P=0.0^a^)
C4C5LAPD	7.80±1.87	42.55 (P=0.0^a^)
C5C6LAPD	7.41±1.9	39.7 (P=0.0^a^)
C6C7LAPD	7.86±1.91	41.86 (P=0.0^a^)
C2C3RCA	59.10±21.18	28.45 (P=0.0^a^)
C3C4RCA	48.88±18.11	25.26 (P=0.0^a^)
C4C5RCA	48.45±15.72	31.42 (P=0.0^a^)
C5C6RCA	46.31±20.53	23 (P=0.0^a^)
C6C7RCA	47.96±18.1	27.02 (P=0.0^a^)
C2C3LCA	59.96±21.76	28.09 (P=0.0^a^)
C3C4LCA	45.98±18.68	25.09 (P=0.0^a^)
C4C5LCA	46.25±18.07	26.1 (P=0.0^a^)
C5C6LCA	44.87±18.6	24.6 (P=0.0^a^)
C6C7LCA	46.96±16.9	28.33 (P=0.0^a^)
C2C3IVDH	5.45±1.07	51.85 (P=0.0^a^)
C3C4IVDH	5.71±1.15	50.6 (P=0.0^a^)
C4C5IVDH	5.56±1.2	47.12 (P=0.0^a^)
C5C6IVDH	5.33±1.44	37.64 (P=0.0^a^)
C6C7IVDH	5.89±1.34	44.81 (P=0.0^a^)
IVDHM	5.59±1.03	55.35 (P=0.0^a^)

## Discussion

This study presents a comprehensive morphometric evaluation of intervertebral foramina and intervertebral disc height in the cervical spine using CT. The analysis revealed statistically significant variations in foraminal dimensions and disc height based on age, gender, and vertebral level, highlighting key anatomical changes that occur with aging and between sexes, which have substantial clinical relevance in cervical spine pathology.

Age-related morphometric changes

The most striking observation from the study is the progressive narrowing of the intervertebral foramina with age, particularly in the VH and CA measurements. For instance, the right vertical height (RVH) at the C6C7 level significantly declined from 12.61±2.2 mm in adults (19-25 years) to 9.86±1.66 mm in elders (>64 years) (one-way ANOVA test=5.52, P-value=0.004). Similarly, the right cross-sectional area (RCA) at C5C6 dropped from 57.7±28.08 mm² in adolescents to 33.6±14.49 mm² in elders (one-way ANOVA test=6.54, P-value=0.001), while at C6C7 it decreased from 58.35±14.42 mm² in adults to 35.77±15.82 mm² in elders (one-way ANOVA test=6.89, P-value=0.0). These results are consistent with previous research indicating that disc degeneration and osteophyte formation contribute significantly to foraminal stenosis [12,15-17].

The level-wise decline in VH and CA across C3C4 to C6C7 reflects progressive degenerative disc disease, which can compress nerve roots and lead to clinical conditions such as cervical spondylosis and radiculopathy [8,15]. These age-related reductions are not uniform across all levels, suggesting that the lower cervical levels are more prone to degenerative changes [18], who emphasized the vulnerability of lower cervical foramina to compressive forces and aging.

Disc height and degeneration

In line with foraminal narrowing, the study also observed a significant decrease in IVDH with advancing age. The average IVDH at the C5C6 level decreased from 6.5±1.6 mm in adolescents to 4.53±1.49 mm in elders (one-way ANOVA test=4.93, P-value=0.003), supporting the association between disc height reduction and degenerative disc disease. The overall IVDHM also showed a significant age-related decline from 6.66±0.92 mm to 5.16±1.04 mm (one-way ANOVA test=3.31, P-value=0.02), reinforcing the understanding that disc degeneration is a key driver of spinal stenosis and nerve root impingement [12,15,18-20].

Gender-based differences

The analysis revealed notable gender differences in cervical foraminal dimensions. Males consistently exhibited significantly larger vertical heights and, to a lesser extent, cross-sectional areas. For example, at the C2C3 level, males had an RVH of 11.52±1.72 mm compared to 10.18±1.83 mm in females (independent-samples t-test=3.83, P-value=0.0), while the left CA at C2C3 was also significantly higher in males (64.74±22.93 mm² vs. 54.39±19.07 mm²; independent-samples t-test=2.47, P-value=0.01). These differences may be attributed to inherent anatomical and biomechanical variations between sexes, such as larger bony structures and more robust musculature in males [11,21-23].

Vertebral level-specific patterns

Across vertebral levels, the lower cervical spine, especially C5C6 and C6C7, demonstrated more pronounced morphometric changes in both foraminal dimensions and disc height. These levels also showed high interlevel correlation in vertical height and cross-sectional area, suggesting a predictable pattern of degeneration [11]. For instance, the right foraminal CA decreased significantly from C2C3 (59.1±21.18 mm², one-sample t-test=28.45, P-value=0.0) to C6C7 (47.96±18.1 mm², one-sample t-test=27.02, P-value=0.0), consistent with the notion that the lower cervical spine endures greater mechanical stress and degenerative load [9,15].

Interestingly, level-wise comparisons revealed that C5C6 and C6C7 levels tend to be more stable in terms of vertical height and cross-sectional area changes, yet they still exhibit the most notable degenerative effects with aging, likely due to cumulative biomechanical loading and wear over time [3,5].

Clinical implications

The findings of this study have important clinical implications. Degenerative narrowing of the intervertebral foramina may lead to cervical radiculopathy, presenting as neck and upper limb pain, numbness, or weakness [24-27]. Most commonly, the C7 root (C6C7 level) is affected, followed by C6 (C5C6) and C8 (C7T1), consistent with both our findings and prior literature [10].

Moreover, radiologists and surgeons must be acutely aware of foraminal anatomy, especially its vascular and neural contents, during interventional procedures to avoid iatrogenic injury [1]. The present data reinforce the need for age-, sex-, and vertebral level-specific considerations during radiologic interpretation and surgical planning for cervical spine disorders.

Limitations of the study

This study has the limitation of a relatively small sample size; therefore, it may be necessary to conduct a larger cohort study to generalize these findings.

## Conclusions

This study reveals a clear pattern of age-related narrowing in intervertebral foraminal dimensions, particularly in vertical height and cross-sectional area, most prominently in the lower cervical spine, such as C5C6 and C6C7. Moreover, the observed gender-based differences, where males consistently exhibited larger foraminal dimensions and disc heights, underscore the importance of considering sex-specific anatomy in clinical assessments. While most intervertebral foraminal dimensions were symmetrical, slight asymmetry at certain levels, such as C4C5, suggests potential implications for side-dominant symptomatology. Future research incorporating clinical correlation with symptoms and outcomes, as well as comparisons with MRI findings, would further strengthen our understanding of cervical spine anatomy and its associated pathologies.
